# Mild hyperbaric oxygen enhances recovery of the plantaris muscle atrophy induced by cast immobilization of the hindlimb in male rats

**DOI:** 10.14814/phy2.70350

**Published:** 2025-05-05

**Authors:** Ai Takemura, Tatsuro Egawa, Ryo Takagi, Ryota Iyama, Zhao Haiyu, Shinichiro Suzuki, Reika Fujino, Takuya Fukunaga, Tatsuya Hayashi, Satoshi Fujita

**Affiliations:** ^1^ Ritsumeikan Global Innovation Research Organization Ritsumeikan University Shiga Japan; ^2^ Graduate School of Human and Environmental Studies Kyoto University Kyoto Japan; ^3^ School of Nursing and Rehabilitation Sciences Showa University Kanagawa Japan; ^4^ Faculty of Sport and Health Science Ritsumeikan University Shiga Japan

**Keywords:** cast immobilization, mild hyperbaric oxygen, mitochondria, muscle atrophy, recovery

## Abstract

Loss of muscle mass is associated with muscle functional decline and mortality. The present study aimed to determine whether exposure to mild hyperbaric oxygen (MHO) during and after casting immobilization reduces muscle atrophy. We distributed eight‐week‐old rats into control (CON), cast immobilization (Cast), and Cast + MHO (1.3 atmosphere absolute with 38% oxygen) groups. Rats were cast for 2 weeks under the normal or MHO condition, followed by a two‐week recovery period under the same condition after cast removal. The plantaris muscle weight (mg/g BW) decreased by approximately 11.5% in the Cast group compared to the CON group (*p* < 0.01), while there were no differences between the CON and Cast + MHO groups, suggesting that MHO enhanced the recovery of muscle atrophy. However, the soleus muscle weight (mg/g BW) decreased by casting immobilization, regardless of MHO. The enzyme activity by succinate dehydrogenase (SDH) staining in the plantaris muscle was lower in the Cast group than in the CON group (*p* < 0.01), while there were no differences between the CON and Cast + MHO groups. In summary, MHO enhances the recovery of plantaris muscle atrophy and partially attenuates the decreased SDH activity after cast immobilization of hindlimb in rats.

## 1 INTRODUCTION

Muscle atrophy is induced by several factors, including long‐term immobilization (casting and bed rest), diseases such as diabetes and cancer, or aging (Bonaldo & Sandri, 2013). Loss of skeletal muscle mass is associated with muscle functional decline, frailty, and mortality (Xu et al., 2022). In rodents, hindlimb casting immobilization and unloading have been used as disuse muscle atrophy models (Gao et al., 2018).

A previous study showed that immobilization induced mitochondrial down‐regulation and increased protein ubiquitination during the early phases, 48 hours after immobilization, in human skeletal muscle (Abadi et al., 2009). Overexpression of peroxisome proliferator‐activated receptor‐γ coactivator‐1α (PGC‐1α), a critical regulator of mitochondrial biogenesis in muscle, prevented disuse‐induced atrophy in rodents (Brault et al., 2010; Sandri et al., 2006; Wang et al., 2017). Muscle‐specific PGC‐1α/β knockout impaired the ability to recover strength following disuse; hind limb unloading (Trevino et al., 2019). PGC‐1α prevents the transcriptional activity of forkhead box (FOX) O3a, which induces the expression of the atrophy‐related ubiquitin ligases atrogin‐1 and muscle RING finger protein‐1 (MuRF‐1) and skeletal muscle atrophy (Sandri et al., 2006). These previous studies showed that PGC‐1α in skeletal muscle plays an essential role in preventing muscle atrophy (Petrocelli & Drummond, 2020).

Mild hyperbaric oxygen (MHO) is an environment of 1.25–1.30 atmosphere absolute (ATA) with 36–40% oxygen; it has been shown to ameliorate muscle atrophy and enhance oxidative metabolism of skeletal muscles in muscle disuse or a model of diabetes and metabolic syndrome rats (Fujita et al., 2012; Takemura et al., 2017; Takemura & Ishihara, 2017). The MHO environment reduced muscle atrophy induced by hindlimb unloading concomitant with increased Pgc‐1α mRNA expression and reduced FOXO1 mRNA expression (Takemura et al., 2017). The unloading‐induced intervention has been used as a microgravity stimulation model, which mimics spaceflight adaptation on cardiovascular and immune systems (Morey‐Holton & Globus, 2002). This study uses the cast immobilization method as a skeletal muscle atrophy model because it simulates clinically relevant inactivity more closely.

The present study aimed to determine whether exposure to MHO during and after casting immobilization enhances the recovery from muscle atrophy. We hypothesized that exposure to MHO alleviated the reduction of mitochondria‐related protein levels and muscle atrophy induced by cast immobilization.

## 2 METHODS

### 2.1 Ethics approval

All experimental and animal care procedures followed the Guidelines for the Care and Use of Laboratory Animals issued by the Institutional Animal Experiment Committee of Kyoto University (22‐A‐5).

### 2.2 Experimental animals and treatments

**FIGURE 1:**
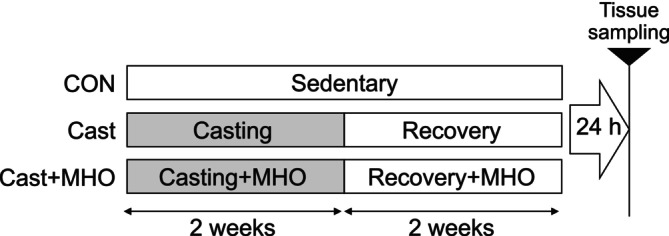
Experimental procedure. In the Cast and Cast + MHO groups, rats were cast for 2 weeks under normal or MHO conditions and removed from the cast. After the cast removal, rats were maintained under normal or MHO conditions during a two‐week recovery period in the Cast and Cast + MHO groups. CON, control; Cast, cast immobilization; Cast + MHO, cast immobilization with mild hyperbaric oxygen.

We randomly distributed eight‐week‐old male Sprague Dawley rats (Simizu Laboratory Animal Supply, Kyoto, Japan) into control (CON, *n* = 8), cast immobilization (Cast, *n* = 7), and cast immobilization + mild hyperbaric oxygen (Cast + MHO, *n* = 7) groups (Figure 1). Casting immobilization of the hindlimb was performed in the Cast and Cast + MHO groups as reported (Tomiya et al., 2019). Briefly, both hindlimbs were fixed in a standard position with casting tape (Scotchcast Plus J; 3 M Health Care, Saint Paul, MN, USA) under anesthesia. In the Cast and Cast + MHO groups, rats were cast for 2 weeks under the normal or MHO conditions, and we subsequently removed the cast. After the cast removal, rats were maintained under normal or MHO conditions over a two‐week recovery period in the Cast and Cast + MHO groups. Rats were housed individually under one ATA with 20.9% oxygen (normobaric conditions) for 4 weeks. Rats in the Cast + MHO groups were exposed to 1.3 ATA with 38% oxygen for 3 h/day (11:30–14:30 h) using a chamber for 4 weeks. Food (MF; Oriental Yeast, Tokyo, Japan) and water were provided ad libitum to all the groups. The room was maintained in a controlled 12‐h light/dark cycle (dark period from 20:00 to 08:00 h) at 22°C ± 2°C and 45–55% relative humidity. The soleus and plantaris were removed from each rat under anesthesia 24 hours after the final MHO intervention, weighed, rapidly frozen in liquid nitrogen, and stored at −80°C until further analysis.

### 2.3 Western blotting

The left soleus and plantaris muscles were collected, rapidly frozen in liquid nitrogen, and stored at −80°C until further analysis. The frozen skeletal muscles were homogenized in radioimmunoprecipitation assay lysis buffer (20–188, Millipore, MA, USA) containing a protease inhibitor (1,183,617,001, Complete Mini EDTA‐free, Roche Life Science, Indianapolis, IN, USA) and phosphatase inhibitor mixture (04906837001, PhosSTOP phosphatase inhibitor cocktail, Roche Life Science). The protein concentrations of the samples were determined using a BCA Protein Assay Kit (23,227, Pierce, Rockford, IL, USA). Protein samples (10 μg) were electrophoresed on 12% sodium dodecyl sulfate‐polyacrylamide gels for 40 min at 200 V. The proteins were transferred from the gels to polyvinylidene difluoride membranes. The membranes were then blocked at room temperature with Blocking One and One‐P (03953–66 and 05999–84, respectively, Nakalai Tesque, Kyoto, Japan) for five and 20 min, respectively. The membranes were incubated with the following primary antibodies: anti‐oxidative phosphorylation (OXPHOS, mouse monoclonal antibody: mAbs, ab110413, Abcam, Cambridge, UK), anti‐PGC‐1α (rabbit polyclonal antibody: pAb, 516,557, Millipore), vascular endothelial growth factor (VEGF, mouse mAb, sc‐7269, Santa Cruz, TX, USA), total‐dynamin‐related protein 1 (Drp1, mouse mAb, ab56788, Abcam, Cambridge, UK), phospho‐Drp1 (Ser616, rabbit pAb, 3455S, CST), fission, mitochondrial 1 (Fis1, rabbit pAb, ab96764, abcam), OPA1 mitochondrial dynamin like GTPase (Opa1, mouse mAb, 612,606, BD Biosciences, NJ, USA), Mitofusin2 (Mfn2, rabbit mAb, ab124773, abcam) total‐eukaryotic translation initiation factor 4E‐binding protein 1 (4EBP1, rabbit mAb, #9644, CST, MA, USA), phospho‐4EBP1 (Tr37/46, rabbit mAb, #2855, CST), total‐p70 ribosomal S6 kinase (p70S6K, rabbit mAb, #34475, CST), phospho‐p70S6K (Thr389, rabbit mAb, #9234, CST), total‐ribosomal protein S6 (rpS6, rabbit mAb, #2217, CST), phospho‐rpS6 (Ser240/244, rabbit pAb, #2215, CST), MuRF‐1 (rabbit mAb, ab172479, Abcam), Atrogin‐1 (rabbit mAb, ab168372, Abcam), and ubiquitinated proteins (mouse mAb, #3936, CST). Membranes were then incubated for 60 min at room temperature with the following secondary antibodies: goat anti‐rabbit IgG (HRP‐linked antibody, #7074, CST) and horse anti‐mouse IgG (HRP‐linked antibody, #7076, CST). After detection of the bands, antibodies were stripped using a stripping solution (193–16,375, Wako, Osaka, Japan) for 10 min at room temperature.

The proteins were detected using Immobilon Forte Western HRP substrate (WBLUF0100, Millipore) and visualized using a FUSION Chemiluminescence Imaging System (M&S Instruments, Osaka, Japan). Band intensities were quantified using Image J software (National Institutes of Health, Bethesda, MD, USA). Two bands were detected and quantified corresponding to Opa1. For total 4E‐BP1, the upper (γ), middle (β), and lower (α) bands were quantified and γ/total α + β + γ was calculated. Three bands were detected and quantified corresponding to phospho‐4E‐BP1. Blots of ubiquitin conjugated were quantified in the range between 25 and 150 kDa. The intensity of Ponceau‐S staining (25–150 kDa, P7170‐1 L, Sigma‐Aldrich) was used as a loading control as previously described (Kotani et al., 2024; Peker et al., 2018).

### 2.4 Histochemical analyses

For succinate dehydrogenase (SDH) staining, the right soleus and plantaris muscles were removed and rapidly frozen in isopentane cooled with liquid nitrogen. These muscles were mounted with an optimal cutting temperature compound (Sakura Finetek, Tokyo, Japan). Transverse 10‐μm slices were sectioned with a cryostat at −20°C, air‐dried, and stored at −20°C. The SDH staining was performed as previously described (Takemura & Ishihara, 2017). Briefly, tissue slides were incubated in a solution containing succinate (224,731, Sigma, MO, USA, 16 mg/mL) and nitroblue tetrazolium (144–01993, Wako, 1 mg/mL) in 0.2 M phosphate buffer (pH 7.5) for 15 min at 37 °C. SDH density was measured using Image J software (NIH). Gray levels were measured at each pixel; gray level 0 was equivalent to 100% light transmission, whereas gray level 255 was equivalent to 0% light transmission. The average intensity of all pixels in the whole muscle image was determined. The cross‐sectional area (CSA) of approximately 300 fibers (3 regions of 100 fibers) of the muscle was determined.

### 2.5 Statistical analysis

Data are expressed as mean ± standard deviation (SD). Differences between the CON, Cast, and Cast + MHO groups were evaluated using a one‐way analysis of variance, followed by the Tukey–Kramer multiple‐comparison test. All statistical analyses were performed using the GraphPad Prism software (Ver. 9.0, Macintosh, GraphPad Software, La Jolla, CA). Statistical significance was defined as *p* < 0.05.

## 3 RESULTS

### 3.1 Muscle weights

**FIGURE 2:**
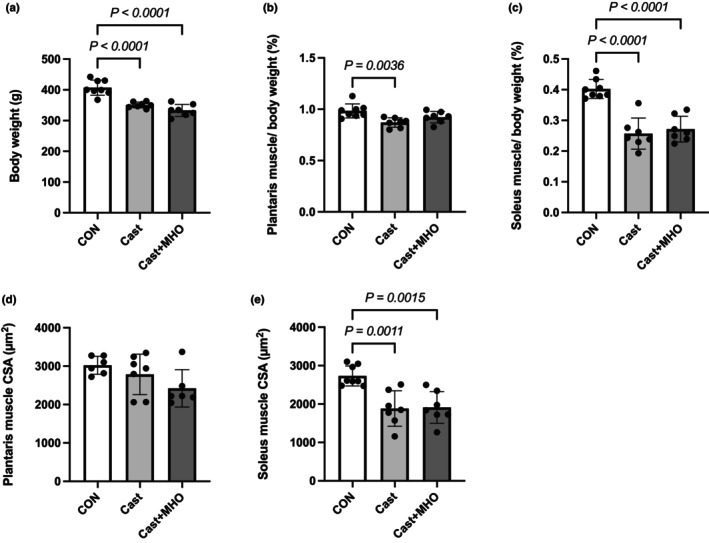
Effects of mild hyperbaric oxygen (MHO) on the body weight (a), plantaris muscle weight (%body weight, b), soleus muscle (%body weight, c), plantaris cross‐sectional area (CSA, d), and soleus CSA (e) in rats. Values are presented as means ± SD (*n* = 7–8). CON, control; Cast, cast immobilization; Cast + MHO, cast immobilization with mild hyperbaric oxygen.

After the intervention period (Figure 1), body weight was lower in the Cast and Cast + MHO group than in the CON group (*p* < 0.0001) (Figure 2a). The plantaris muscle weight (mg/g BW) was lower in the Cast group than in the CON group (*p* < 0.01), while there were no significant differences between the CON and Cast + MHO groups (Figure 2b). The soleus muscle weight (mg/g BW) was lower in the Cast and Cast + MHO group than in the CON group (*p* < 0.0001) (Figure 2c). There were no differences in plantaris muscle CSA among groups (Figure 2d). The soleus CSA was lower in the Cast and Cast + MHO group than in the CON group (*p* < 0.01) (Figure 2e).

### 3.2 Mitochondria‐related proteins and enzyme activity

**FIGURE 3:**
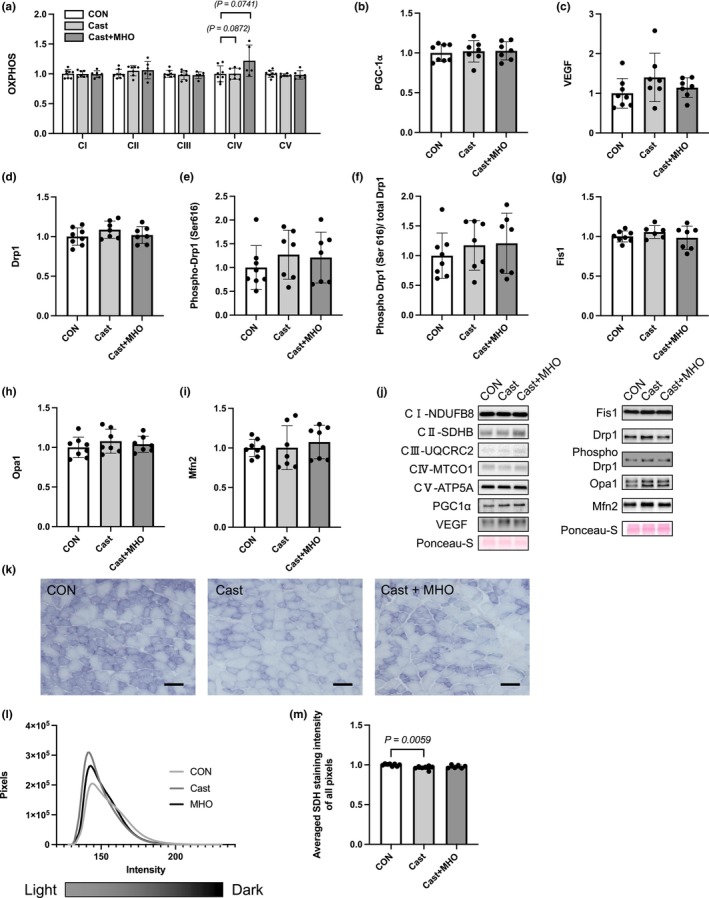
Effects of mild hyperbaric oxygen (MHO) on the mitochondrial proteins (a) including complex I; NDUF88, complex II; SDHB, complex III; UQCRX2, complex IV; MTCO1, and complex V; ATP5A, PGC‐1α (b), VEGF (c), Drp1 (d), Phospho‐ Drp1 (e), Phosphorylation ratio of Drp1 (f), Fis1 (g), Opa1 (h) and Mfn2 (i) protein levels in plantaris muscle in rats. Representative western blots (j) in plantaris muscle. Transverse sections stained for succinate dehydrogenase (SDH) (k), the number of pixels at each SDH intensity (l), and averaged SDH staining intensity of all pixels in the whole muscle (m). Values are presented as means ± SD (*n* = 7–8). CON, control; Cast, cast immobilization; Cast + MHO, cast immobilization with MHO. Bar represents 100 μm.

There were no differences in the levels of mitochondrial proteins (complex I; NDUFB8, complex II; SDHB, complex III; UQCRC2, complex IV; MTCO1, complex V; ATP5A), PGC‐1α, VEGF proteins, or mitochondria dynamics proteins (Drp1, Fis1, Opa1, and Mfn2) expression in the plantaris muscle between the three groups (Figure 3a–j). The levels of complex IV of mitochondrial proteins in plantaris muscle in the Cast + MHO group tended to be higher than those in the CON and Cast groups (*p* = 0.0741 and 0.0872, respectively) (Figure 3a,h). The enzyme activity by SDH staining in the plantaris muscle in the Cast group was significantly lower than the CON group (*p* < 0.01). At the same time, there were no differences between the CON and Cast + MHO groups (Figure 3k–m).

**FIGURE 4:**
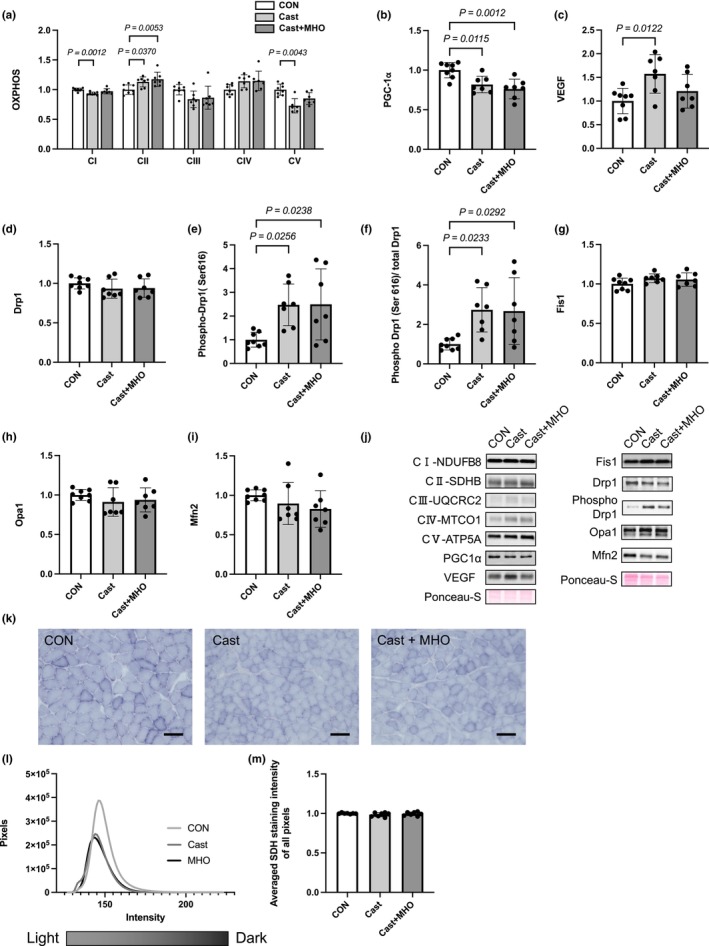
Effects of mild hyperbaric oxygen (MHO) on the mitochondrial proteins (a) including complex I; NDUF88, complex II; SDHB, complex III; UQCRX2, complex IV; MTCO1, and complex V; ATP5A, PGC‐1α (b), VEGF (c), Drp1 (d), Phospho‐ Drp1 (e), Phosphorylation ratio of Drp1 (f), Fis1 (g), Opa1 (h) and Mfn2 (i) protein levels in soleus muscle in rats. Representative western blots (j) in soleus muscle. Transverse sections stained for SDH (k), the number of pixels at each SDH intensity (l), and averaged SDH staining intensity of all pixels in the whole muscle (m). Values are presented as means ± SD (*n* = 7–8). CON, control; Cast, cast immobilization; Cast + MHO, cast immobilization with MHO. Bar represents 100 μm.

The levels of complex I of mitochondrial proteins in the Cast group were lower than those in the CON group in the soleus muscle (*p* < 0.01), while there were no differences in it between the CON and Cast + MHO groups (Figure 4a,j). The levels of complex II of mitochondrial proteins in the Cast and Cast + MHO groups were higher than those in the CON group in the soleus muscle (*p* < 0.05 and 0.01, respectively) (Figure 4a,j). There were no significant differences in the levels of complex III and IV of mitochondrial proteins in the soleus muscle between the three groups (Figure 4a,j). The levels of complex V of mitochondrial proteins in the Cast group were lower than those in the CON group in the soleus muscle (*p* < 0.01), while there were no differences in it between CON and Cast + MHO groups (Figure 4a,j). The levels of PGC‐1α in the Cast and Cast + MHO groups were lower than those in the CON group in the soleus muscle (*p* < 0.05 and 0.01, respectively) (Figure 4b,j). The levels of VEGF in the Cast group were higher than those in the CON group in the soleus muscle (*p* < 0.05) (Figure 4c,j). There were no significant differences in the levels of total‐Drp1 in the soleus muscle between groups (Figure 4d,j). The levels of phospho‐Drp1 and the phosphorylation ratio of Drp1 that relate to mitochondrial fission in the Cast and Cast + MHO groups were higher than those in the CON group in the soleus muscle (*p* < 0.05) (Figure 4e,f,j). There were no significant differences in the Fis1, Opa1, and Mfn2 levels in the soleus muscle between the three groups (Figure 4g–j). There were no significant differences in the enzyme activity by SDH staining in the soleus muscle between the three groups (Figure 4i–k).

### 3.3 Anabolic signal‐related proteins

**FIGURE 5:**
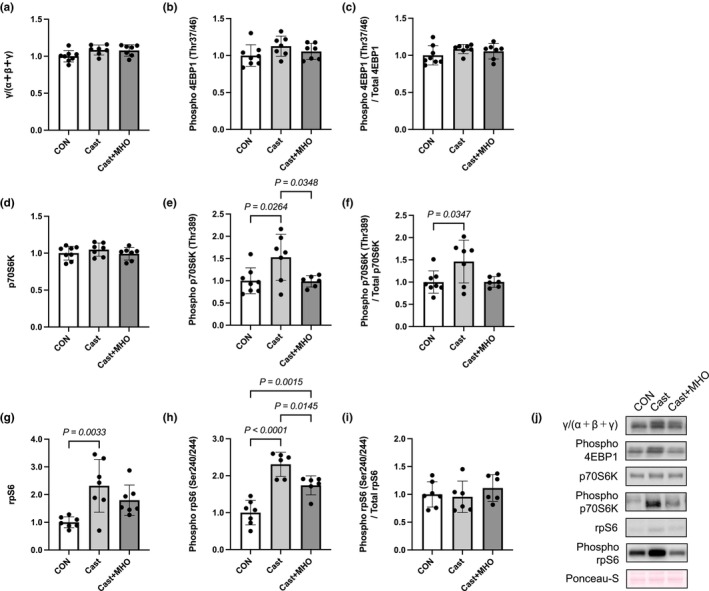
Effects of mild hyperbaric oxygen (MHO) on the anabolic proteins in plantaris muscle. The total (a) and phosphorylation (b) protein levels and phosphorylation ratio (c) of 4EBP1. The total (d) and phosphorylation (e) protein levels and phosphorylation ratio (Thr389, f) of p70S6K. The total (g) and phosphorylation (h) protein levels and phosphorylation ratio (Ser240/244, i) of rpS6. Representative western blots (j) in plantaris muscle. Values are presented as means ± SD (*n* = 7–8). CON, control; Cast, cast immobilization; Cast + MHO, cast immobilization with MHO.

There were no differences in the total (γ/total α + β + γ) and phosphorylation levels and phosphorylation ratio of 4E‐BP1 and total level of p70S6K in the plantaris muscle between the three groups (Figure 5a–d,j). The phosphorylation levels of p70S6K in the plantaris muscle in the Cast group were significantly higher than those in the CON and Cast + MHO groups (*p* < 0.05) (Figure 5e,j). The phosphorylation ratio of p70S6K and the total level of rpS6 in the plantaris muscle in the Cast group were significantly higher than those in the CON groups (*p* < 0.05 and 0.01, respectively). At the same time, there were no differences between the CON and Cast + MHO groups (Figure 5f,g,j). The phosphorylation level of rpS6 in the plantaris muscle in the Cast group was significantly higher than in the CON and Cast + MHO groups (*p* < 0.0001 and 0.05, respectively). Those in the Cast + MHO group were higher than those in the CON group (*p* < 0.01) (Figure 5h,j). There were no differences in the phosphorylation ratio of rpS6 in the plantaris muscle between the three groups (Figure 5i,j).

**FIGURE 6:**
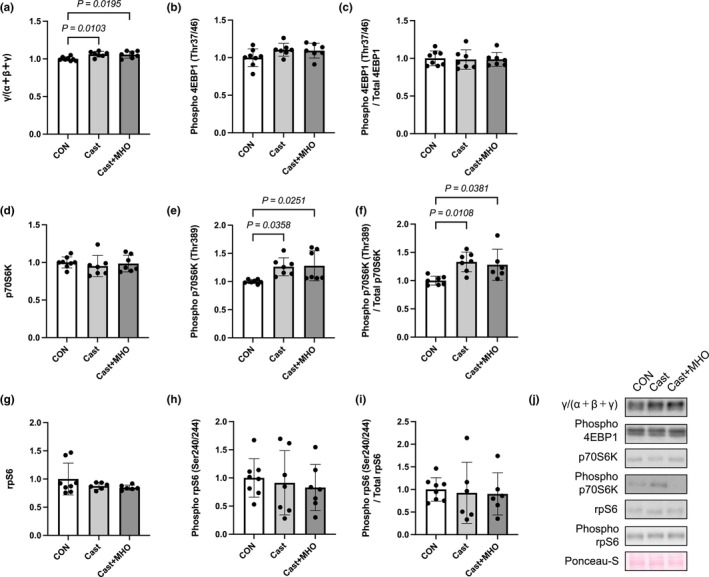
Effects of mild hyperbaric oxygen (MHO) on the anabolic proteins in soleus muscle. The total (a) and phosphorylation (b) protein levels and phosphorylation ratio (c) of 4EBP1. The total (d) and phosphorylation (e) protein levels and phosphorylation ratio (Thr389, f) of p70S6K. The total (g) and phosphorylation (h) protein levels and phosphorylation ratio (Ser240/244, i) of rpS6. Representative western blots (j) in soleus muscle. Values are presented as means ± SD (*n* = 7–8). CON, control; Cast, cast immobilization; Cast + MHO, cast immobilization with MHO.

The total levels of 4E‐BP1 in the soleus muscle in the CON group were significantly lower than those in the Cast and Cast + MHO groups (*p* < 0.05, respectively) (Figure 6a,j). There were no differences in the phosphorylation levels and the phosphorylation ratio of 4E‐BP1 and the total level of p70S6K in the soleus muscle between the three groups (Figure 6b–d,j). The phosphorylation levels and the phosphorylation ratio of p70S6K in the soleus muscle in the CON group were significantly lower than those in the Cast and Cast + MHO groups (*p* < 0.05) (Figure 6e,f,j). There were no differences in the total and phosphorylation levels and phosphorylation ratio of rpS6 in the soleus muscle between the three groups (Figure 6g–j).

### 3.4 Catabolic signal‐related proteins

**FIGURE 7:**
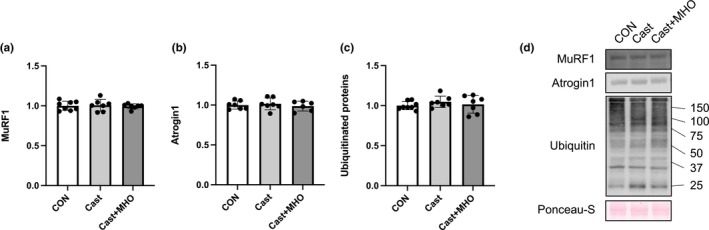
Effects of mild hyperbaric oxygen (MHO) on the catabolic proteins in plantaris muscle. MuRF1 (a), Atrogin1 (b), Ubiquitinated protein (c) levels. Representative western blots (d) in plantaris muscle. Values are presented as means ± SD (*n* = 7–8). CON, control; Cast, cast immobilization; Cast + MHO, cast immobilization with MHO.

**FIGURE 8:**
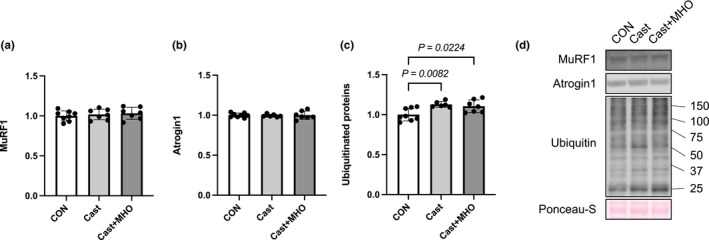
Effects of mild hyperbaric oxygen (MHO) on the catabolic proteins in soleus muscles. MuRF1 (a), Atrogin1 (b), Ubiquitinated protein (c) levels. Representative western blots (d) in soleus muscle. Values are presented as means ± SD (*n* = 7–8). CON, control; Cast, cast immobilization; Cast + MHO, cast immobilization with MHO.

There were no differences in the levels of MuRF1 and Atrogin1 in the plantaris (Figure 7a–d) and soleus muscles and ubiquitinated protein in the plantaris muscle between the three groups (Figure 8a,b,d). The levels of ubiquitinated proteins in the soleus muscle in the CON group were significantly lower than those in the Cast and Cast + MHO groups (*p* < 0.01 and 0.05, respectively) (Figure 8c,d).

## 4 DISCUSSION

We investigated the effects of mild hyperbaric oxygen (MHO) on muscle weight and mitochondrial protein levels after muscle disuse in rats. Notably, MHO was administered both during the period of cast immobilization and throughout the subsequent recovery phase. Our findings indicate that MHO facilitates the recovery of plantaris muscle atrophy and mitigates the reduction in mitochondrial protein levels and succinate dehydrogenase (SDH) activity that occurs after cast immobilization. These results suggest that continuous MHO exposure, spanning both disuse and recovery periods, may play a crucial role in preserving muscle integrity and mitochondrial function during immobilization‐induced muscle atrophy.

A previous study showed that exposing rats to MHO under 1.25 ATA with 36% oxygen for 2 weeks before and 2 weeks after hindlimb unloading increased *Pgc‐1α* mRNA expression (Takemura et al., 2017). Another study demonstrated that mitochondrial biogenesis via PGC‐1α plays an essential role in maintaining muscle weight (Abadi et al., 2009; Petrocelli & Drummond, 2020). Overexpression of PGC‐1α in muscle has been shown to prevent disuse‐induced atrophy in rodents (Brault et al., 2010; Sandri et al., 2006; Wang et al., 2017), whereas muscle‐specific PGC‐1α/β knockout reduced recovery of strength following disuse (Trevino et al., 2019). In a previous study, MHO exposure was applied only after hindlimb unloading, with no exposure during the unloading period, under 1.3 ATA and 36% oxygen for 2 weeks. However, this treatment did not restore soleus SDH activity or mitochondrial content index (Takemura et al., 2017). On the contrary, the present study showed that the MHO condition both during casting immobilization and recovery periods better maintained the mitochondrial content in rats. MHO increased the SDH staining intensity in the plantaris muscle and the levels of mitochondrial complex I and V in the soleus muscle. However, there is no significant difference in mitochondrial biogenesis or dynamics, including PGC‐1α, Drp1, Fis1, Opa1, and Mfn2 in the soleus and plantaris muscles between Cast and Cast + MHO groups in this study. The protein expression level of PGC‐1α decreased and the phosphorylation ratio of Drp1 increased at the reload after disuse regardless of MHO exposure in the soleus muscle. Further studies are needed to elucidate the underlying mechanisms that contributed to the maintenance of mitochondrial content observed under MHO conditions.

A previous study showed that the exposure to MHO, the condition under 1.25 ATA with 36% oxygen for 8 weeks (3 h/day), improved capillary rarefaction and increased capillary diameter and volume in the soleus muscle of sedentary streptozotocin‐induced type 1 diabetic rats (Tanaka et al., 2021). Under the MHO environment under 1.25 ATA and 36% oxygen for at least 4 weeks in a sedentary condition, some studies showed that PGC‐1α level increased (Takemura et al., 2017; Takemura & Ishihara, 2017). High PGC‐1α levels promote angiogenesis via VEGF protein expression (Fujiwara et al., 2023). These previous studies showed that PGC‐1α induced angiogenesis via VEGF and attenuated capillary rarefaction, thereby improving muscle atrophy under MHO conditions. On the other hand, a previous study showed that the reload after the disuse promoted angiogenesis via increased VEGF protein levels to promote the regrowth of myofibers and hypertrophy (Kataoka et al., 2014; Nakano et al., 2009; Plyley et al., 1998). Similar to the previous study, the increased VEGF protein induced by reloading after casting immobilization was shown; however, there are no differences between CON and Cast + MHO groups in the soleus muscle in this study. This contradiction about VEGF protein level may be related to PGC‐1α expression levels, which were not increased by MHO in this study.

This study investigated whether the MHO condition attenuates atrophy during casting immobilization and improves the recovery from atrophy after casting immobilization. Reload, i.e., the recovery period after muscle atrophy following disuse, restored the expression levels of the anabolic protein to levels that exceed those of control conditions (Baehr et al., 2016; Figueiredo et al., 2021; Miller et al., 2019). In this study, the phosphorylation ratio of p70S6K in the soleus and plantaris muscles of the Cast group and the soleus muscle of the Cast + MHO group was increased compared to those of the CON group at 2 weeks after the removal of cast immobilization. However, despite reload, there were no significant differences in the phosphorylation ratio of p70S6K in the plantaris muscle between the CON and Cast + MHO groups. In terms of catabolic insight, a previous study showed that exposing rats to MHO, under 1.25 ATA with 36% oxygen for 2 weeks before and 2 weeks after hindlimb unloading, reduced catabolic protein (*FoxO1* mRNA) expression in unloading muscle atrophy rats (Takemura et al., 2017). Conversely, the previous study also showed that exposure to MHO for 2 weeks only after hindlimb unloading did not alleviate the high catabolic protein level (Takemura et al., 2017). The present study shows no differences between the Cast and Cast + MHO groups in the catabolic proteins including MuRF1, Atrogin1, and ubiquitinated proteins two weeks after casting immobilization. Further studies are warranted to determine the optimal duration and timing of MHO exposure—whether before, during, or after disuse‐induced atrophy—in order to maximize anabolic signaling and minimize catabolic responses.

In this study, the plantaris and soleus muscles relative to body weight were reduced by approximately 10% and 35%, respectively, by casting suspension. A previous study showed that the plantaris and soleus muscles relative to body weight were reduced comparatively by approximately 5% and 30%, respectively, by casting suspension (Madokoro et al., 2018). This difference of adaptation between the plantaris and soleus muscles was explained by the proportion of type IIB fibers, i.e., the plantaris muscle contains a high proportion of type IIB fibers and is less susceptible than the soleus muscle to disuse (Madokoro et al., 2018). A previous study showed that rats exposed to MHO under 1.3 ATA and 36% oxygen only after hindlimb unloading for 2 weeks did not fully recover from soleus muscle atrophy (Takemura et al., 2017). On the contrary, this study showed that MHO conditions, both during casting immobilization and recovery periods, enhanced recovery from muscle atrophy. These findings highlight the potential importance of MHO application during the disuse phase, particularly for muscles that are highly susceptible to atrophy, such as the soleus.

The present study has several limitations. First, while we assessed the effect of MHO during both casting immobilization and the reloading period in the current study, this approach did not allow us to distinguish between the specific effects of MHO on muscle atrophy during immobilization and its potential role in recovery during reloading. Future studies should consider separate experimental conditions to isolate these effects. Second, we could not measure the mitochondrial oxygen consumption rate, which is a direct and reliable indicator of mitochondrial respiratory function. Further analysis of mitochondrial respiration would provide a more comprehensive understanding of mitochondrial adaptations in response to MHO. Additionally, we did not perform a functional assessment of protein synthesis using stable isotope tracers. The use of stable isotope tracers would allow for a clearer delineation of the relative contributions of muscle protein synthesis and degradation in mitigating and recovering from muscle atrophy.

## 5 CONCLUSIONS

MHO under the 1.3 ATA with 38% oxygen for 4 weeks, spanning both disuse and recovery periods, enhances recovery from muscle atrophy of the plantaris muscle. It partially attenuates the decreased muscle mitochondria after cast immobilization of the hindlimb in rats.

## CONFLICT OF INTEREST STATEMENT

The authors declare no conflicts of interest.

## Data Availability

The data that support the findings of this study are available from the corresponding author upon reasonable request.
